# Temporal and spatial variation in bacterial communities of “Jonagold” apple (*Malus* x *domestica* Borkh.) and “Conference” pear (*Pyrus communis* L.) floral nectar

**DOI:** 10.1002/mbo3.918

**Published:** 2019-08-22

**Authors:** Jolien Smessaert, Maarten Van Geel, Christel Verreth, Sam Crauwels, Olivier Honnay, Wannes Keulemans, Bart Lievens

**Affiliations:** ^1^ Laboratory for Fruit Breeding and Biotechnology, Department of Biosystems KU Leuven Leuven Belgium; ^2^ Plant Conservation and Population Biology, Biology Department KU Leuven Leuven Belgium; ^3^ Laboratory for Process Microbial Ecology and Bioinspirational Management, Department of Microbial and Molecular Systems KU Leuven Sint‐Katelijne‐Waver Belgium

**Keywords:** conference, Jonagold, metagenomic analysis, nectar bacteria, pollination, pome fruit

## Abstract

Production of many agricultural crops and fruits strongly depends on pollinators. For instance, pome fruits such as apple and pear are highly dependent on pollination for fruit set, fruit quality, and yield. Nectar is often inhabited by microbes, most often yeasts and bacteria, which may change nectar quality and therefore also affect plant–pollinator interactions. Here, we used high‐throughput 16S ribosomal RNA gene amplicon sequencing to investigate the temporal and spatial variation in bacterial communities in floral nectar of apple and pear. We sampled 15 apple (*Malus* x *domestica* Borkh.) and 15 pear (*Pyrus communis* L.) orchards distributed over the eastern part of Belgium over a timespan of seven days. Nectar bacterial community composition differed strongly among fruit species. Nectar of pear was dominated by Actinobacteria, followed by Proteobacteria and Firmicutes. Apple nectar was strongly enriched in Bacteroidetes, a phylum which until now has been found to be rarely associated with floral nectar. Nectar was dominated by only a few bacterial species, with *Brevibacterium* (Actinobacteria*)* and *Undibacterium* (Proteobacteria) as the most abundant bacteria in pear and apple nectar, respectively. Bacterial richness and diversity were found to fluctuate during flowering, likely due to changing environmental conditions. Additionally, spatial structure in nectar bacterial community composition was found in apple orchards, while this was not the case for pear. Differences in nectar bacterial communities between apple and pear nectar may differently affect the chemical and nutritional composition of the nectar, influencing pollinator attraction and visitation, and thus pollination efficacy in general.

## Graphical abstract

Nectar bacterial community composition was dominated by only a few bacterial species and differed strongly among fruit species. The nectar of pear (b) was dominated by Actinobacteria, Proteobacteria, and Firmicutes, while apple nectar (a) was strongly enriched by Bacteroidetes. Bacterial richness and diversity were found to fluctuate during flowering.

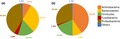

## 1 INTRODUCTION

Production of most agricultural crops and fruits strongly depends on bees or other animals for pollination (Klein et al., 2007), an ecosystem service strongly influenced by floral nectar and pollen (Knauer & Schiestl, 2015; Somme et al., 2015). Recent surveys have shown that nectar is often inhabited by microorganisms, most often yeasts and bacteria, which may change nectar quality and therefore also affect plant–pollinator interactions (reviewed in Pozo, Lievens, & Jacquemyn, 2015). Communities of nectar‐inhabiting microorganisms are generally species‐poor and dominated by a few taxa that are able to survive and thrive in the harsh environment of nectar (Lievens et al., 2015). Most attention so far has been given to nectar yeasts, and the most commonly encountered yeast species include the nectar specialists *Metschnikowia gruessii* and *M. reukaufii* (Pozo et al., 2015). By contrast, only a few studies have focused on the bacterial communities that inhabit floral nectar. Furthermore, most attention has been given to wild plants (Álvarez‐Pérez, Herrera, & de Vega, 2012; Jacquemyn, Lenaerts, Brys, et al., 2013; Jacquemyn, Lenaerts, Tyteca, & Lievens, 2013), whereas few studies have focused on nectar bacteria of agricultural crops (but see Fridman, Izhaki, Gerchman, & Halpern, 2012; Pusey, Stockwell, & Mazzola, 2009; Schaeffer, Vannette, Brittain, Williams, & Fukami, 2017). In general, nectar bacteria belong to a limited set of phyla, including Actinobacteria, Firmicutes, and Proteobacteria (Pozo et al., 2015), although in some nectars also Bacteroidetes were found (Fridman et al., 2012). It has also been found that microbial communities of floral nectar may differ among plant species (Pozo et al., 2015), populations of the same species (Jacquemyn, Lenaerts, Brys, et al., 2013), or even among different flowers of the same plant (Canto & Herrera, 2012), suggesting that not only plant features but also local or temporal conditions affect the community composition.

Microbial metabolism has been shown to affect nectar chemistry, which in turn may have an impact on pollinator foraging and pollination success. More specifically, the presence of microorganisms in floral nectar has been shown to decrease sugar concentrations, alter sugar composition, influence acidity, reduce concentrations of secondary metabolites, and change amino acid content and concentration (Pozo et al., 2015). The metabolic activity of nectar‐inhabiting microorganisms also affects other floral features, including nectar temperature (Herrera & Pozo, 2010) and production of volatiles or fermentation products (Rering, Beck, Hall, McCartney, & Vannette, 2017; Sobhy et al., 2018). An increase in foraging activity of pollinators has been seen when yeasts were present in nectar (Herrera, Pozo, & Medrano, 2013; Schaeffer & Irwin, 2014; Schaeffer, Phillips, Duryea, Andicoechea, & Irwin, 2014). Especially, bumblebees will forage longer on flowers colonized by yeasts and hence remove more nectar which may enhance pollination and, by consequence, crop yield (Schaeffer et al., 2014). On the other hand, there are also studies which indicate that certain pollinators are more likely to reject nectar that has been colonized by bacteria (Good, Gauthier, Vannette, & Fukami, 2014; Junker, Romeike, Keller, & Langen, 2014; Vannette, Gauthier, & Fukami, 2013).

The aim of this study was to gain more insight into the nectar bacterial communities of pome fruit trees. Most pome fruit trees require pollination, and poor pollination results in reduced yield and misshapen fruits (Garratt et al., 2014; Geslin et al., 2017). In order to improve pollination, it is important to know whether nectar microbes inhabit the floral nectar of pome fruit trees, and which nectar microbes occur. Although a few studies have investigated the flowers from apple and pear for microbial presence, often in relation to the fire blight‐causing pathogen *Erwinia amylovora* (Pusey et al., 2009; Shade, McManus, & Handelsman, 2013), no study so far focused specifically on the microorganisms inhabiting their nectar.

Here, using high‐throughput sequencing of 16S ribosomal RNA (rRNA) gene amplicons we investigated the bacterial community composition in the floral nectar of “Jonagold” apple and “Conference” pear trees in 30 commercial orchards in Belgium. We addressed the following research questions: (a) Does the bacterial community composition of floral nectar of “Jonagold” apple and “Conference” pear differ among each other; (b) does the nectar bacterial community composition change during the flowering period; (c) are these temporal changes in nectar bacterial community composition fruit species dependent; and (d) is similarity in the nectar bacterial communities among different orchards related to geographical distance? Apart from new fundamental insights, our study provides basic information that can pave the way for specific manipulations to increase pollinator attraction and improve the pollination process.

## 2 MATERIALS AND METHODS

### 2.1 Study area and nectar sampling

The study was carried out in the eastern part of Belgium in 30 commercial apple (*Malus* x *domestica* Borkh.) and pear (*Pyrus communis* L.) orchards, owned by 15 fruit growers. For each grower, both a “Jonagold” apple (further referred to as “apple”) and a “Conference” pear (further referred to as “pear”) orchard were included in the study. The distance between both orchards from the same grower was generally less than 1 km. Orchards from different growers were located at different distances from each other, ranging between 2 and 53 km (Table S1). Nectar samples were taken on three days in 2017 during the main flowering period of pear and apple between the 4 April and the 10 April, and from the 12 April until the 18 April, respectively. At the start of the flowering period (further referred to as “day 1”), all 30 orchards were sampled. Additionally, three days later (further referred to as “day 4”), and again three days later (further referred to as “day 7”), a subset of five apple and five pear orchards belonging to the same grower were sampled (the same pear and apple orchards for both time points). At each time point, samples were collected from the same four pear and the same four apple trees per orchard. Nectar was collected from three randomly selected open flowers per tree as described previously (Lenaerts et al., 2016). Briefly, nectar was collected using 5‐µl microcapillary tubes (Hirschmann Laborgeräte GmbH & Co. KG), and subsequently pooled per tree (resulting in four samples per orchard per time point). To avoid age effects, particular care was taken at each time point to select flowers of the same age (i.e., 1 day after anthesis). Samples were stored at −20°C until further analysis.

### 2.2 DNA extraction, PCR amplification, and metagenomic analysis

For each nectar sample (*c*. 5 µl), genomic DNA was extracted using the phenol–chloroform extraction method described by Lievens et al. (2003). Additionally, a negative control was included during extraction for which the same protocol was followed, but without addition of nectar. DNA samples were then subjected to PCR amplification and sequencing of the V4 region of the bacterial 16S rRNA genes using the Illumina MiSeq sequencing platform. Again a negative control was included (PCR amplification control), this time by replacing template DNA with sterile water. Samples were amplified using sample‐specific barcode‐labeled versions of the primer set 515F/ 806R (Caporaso et al., 2011; dual‐index sequencing strategy, Kozich, Westcott, Baxter, Highlander, & Schloss, 2013; Table S2). Each amplification was performed in a volume of 40 μl containing 1× Titanium Taq PCR buffer, 150 μM of each dNTP, 0.5 μM of each primer, 1× Titanium Taq DNA polymerase (Clontech), and 2 µl 10 times diluted DNA. The reaction was initiated by denaturation at 94°C for 120 s, followed by 30 cycles of denaturation at 94°C for 45 s, annealing at 59°C for 45 s and elongation at 72°C for 45 s, and terminated by a final elongation at 72°C for 10 min. Amplicons were then purified using Agencourt AMPure XP magnetic beads (Beckman Coulter Genomics GmbH) according to the manufacturer's instructions. Following quantification of the purified amplicons using a Qubit High Sensitivity Fluorometer kit (Invitrogen), amplicons were combined at equimolar concentrations into an amplicon library. For both types of negative controls, no PCR amplification was observed (neither by gel electrophoresis nor by amplicon quantification using the Qubit assay), and they were therefore not included in the library. The library was subjected to ethanol precipitation and loaded on agarose gel. Next, the target band (*c*. 400 bp) was excised and the DNA was purified again, this time using the QIAquick Gel Extraction Kit (Qiagen). Finally, the library was diluted to 2 nM and sequenced using an Illumina MiSeq sequencer with v2 500 cycle reagent kit (Illumina).

Sequences were received as a de‐multiplexed FASTQ file (data deposited in the Sequence Read Archive; BioProject accession PRJNA488015 ). Paired‐end reads were merged using USEARCH (v. 10.0.240) to form consensus sequences (Edgar, 2013) with no more than five mismatches allowed in the overlap region. Following removal of the primer sequences, consensus sequences were truncated at the 250th base. Shorter reads or reads with a total expected error threshold above 0.05 for all the bases were discarded. The “classify.seqs” and “remove.lineage” commands in Mothur (v1.39.3) and the Silva database (v1.23) were used to identify and remove potential mitochondrial, chloroplast, archaeal, and eukaryote contaminants. Next, sequences were grouped into operational taxonomic units (OTUs) based on a 3% sequence dissimilarity cutoff using the UPARSE algorithm in USEARCH, during which chimeric sequences were also removed (Edgar, 2013). Subsequently, the dataset was limited to those OTUs representing more than 1.0% of the sequence reads in any sample (i.e., 327 OTUs), and the number of sequences was rarefied to 850, leaving 130 samples in the dataset (*n* = 41 and *n* = 35, *n* = 10 and *n* = 20, and *n* = 8 and *n* = 16 at day 1, day 4, and day 7, for apple and pear, respectively). The taxonomic classification of each OTU was determined with the SINTAX algorithm implemented in USEARCH, (Edgar, 2013) based on the Silva Living Tree Project v1.23 database. Based on such short sequences, taxonomic assignments are generally considered reliable from domain to genus when bootstrap confidence values exceed 0.80. Furthermore, to verify the identity of the most important OTUs, BLAST searches were performed in GenBank against type materials and sequence entries related to the nectar environment (search was limited to sequences associated with the keyword "nectar").

### 2.3 Statistical analyses

For each sample, bacterial OTU richness (*S*) and the Shannon diversity index (*H*) were calculated (Shannon, 1948). The Shannon index was exponentially transformed (Exp(*H*)) to obtain a diversity estimate which behaves in a linear fashion (Jost, 2006). We used a generalized linear model (GLM) to relate OTU richness and diversity to fruit species (factor, two levels) and sampling time (factor, three levels), and their interaction. GLM was chosen instead of generalized repeated measurements as only a subset of the orchards (5 from the 15) were sampled over the three time points and different flowers were monitored over time. We fit Poisson distributed models and included orchard as random factor in the models. When the sampling time x species interaction was significant, the analyses were redone for each species separately to evaluate the effect of sampling time. OTU richness and diversity values were displayed as average ± standard deviation, unless otherwise stated. All analyses were performed in JMP Pro 13 (SAS Institute).

To evaluate differences in bacterial community composition, we performed a redundancy analysis (RDA) based on the sample‐OTU relative abundance matrix (Legendre & Gallagher, 2001) in the R‐package Vegan (R Development Core Team). Differences in nectar bacterial communities between both fruit species and time points were tested for significance based on a permutation test with 1,000 iterations for which orchard was included as random factor in the model. When the sampling time x species interaction was significant, the RDA was redone for each fruit species separately with Bonferroni adjustments. Dispersion ellipses using the standard deviation of the mean were plotted on the ordination representing communities belonging to different sampling times. Furthermore, a Mantel test was conducted for both fruit species together and for each species separately, to test whether bacterial communities were related to geographic distances.

## 3 RESULTS

### 3.1 Bacterial OTU richness and Exp(H) diversity

**Figure 1:**
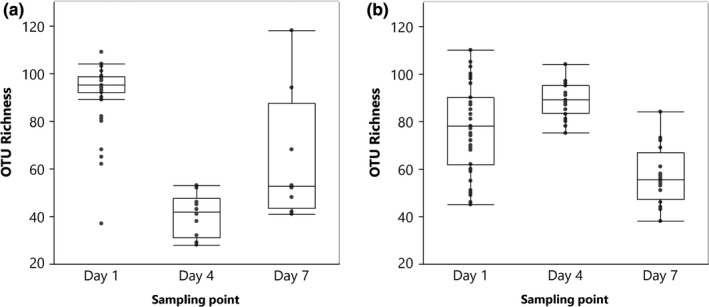
Bacterial OTU richness (*S*) in floral nectar of apple (a) (59 samples) and pear (b) (71 samples) over three time points, including start of the flowering period (day 1), peak blooming (day 4), and three days later (day 7). Outlier box plots represent the distribution of the samples around the median (horizontal line within the box). The first and third quartiles are the ends of the box

**Figure A1:**
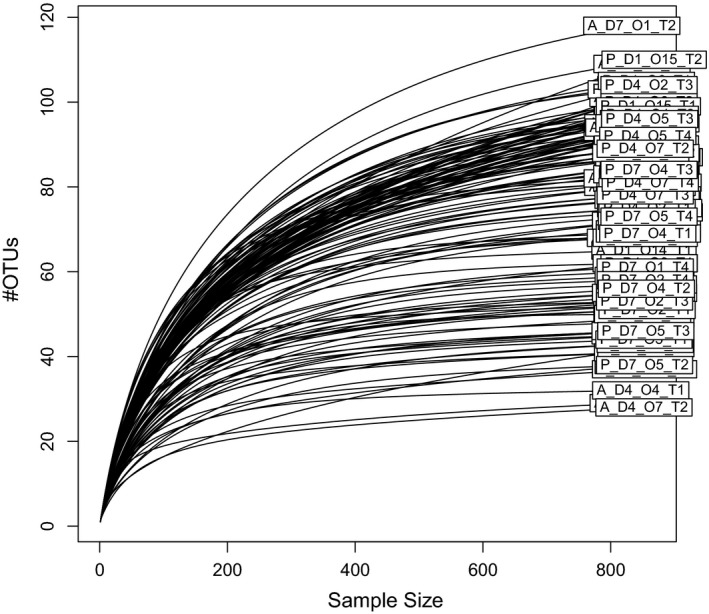
Rarefaction curves showing the number of bacterial operational taxonomic units (OTUs) per sample (A: apple; P: Pear; D: day; O: orchard)

Deep sequencing of 16S rRNA gene amplicons and subsequent bioinformatics analysis resulted in a total of 327 bacterial OTUs (only OTUs representing more than 1.0% of the sequence reads in any sample were retained) (Table S3). Of these, 269 OTUs were found in both apple and pear nectar, while 14 OTUs only occurred in the nectar of pear and 44 OTUs only in the nectar of apple. In general, rarefaction curves tended to approach saturation (Figure A1), indicating that the bacterial communities could be accurately compared at a sequence depth of 850 sequences. OTU richness per sample was similar for apple and pear, and varied between 28 and 118 (average: 79.3 ± 24.9) and between 38 and 110 (average: 76.2 ± 18.4) for apple and pear, respectively. The GLM revealed that for the OTU richness the sampling time x species interaction was significant (*χ* ^2^ = 265.3, *df*: 2, *p* < .0001; Table 1, Figure 1). Splitting the dataset for apple and pear showed that, both for apple and pear, there was a significant effect of sampling time (*χ* ^2^ = 282.5, *df*: 2, *p* < .0001; *χ* ^2^ = 141.2, *df*: 2, *p* < .0001, respectively). For apple, OTU richness at day 1 (91.6 ± 13.1) was significantly higher compared to days 4 (40.7 ± 8.9) and 7 (64.5 ± 27.7) (*p* < .0001, *p* < .0001, respectively). Furthermore, OTU richness at day 4 was significantly lower in comparison with day 7 (*p* < .0001). For the nectar of pear, the highest OTU richness was observed on day 4 (88.9 ± 7.2), which differed significantly from day 1 (77.6 ± 18.2) and day 7 (57.1 ± 12.3) (*p* = .02, *p* < .0001, respectively). Furthermore, OTU richness at day 1 was significantly higher compared to day 7 (*p* < .0001).

**Table 1:** Results of statistical tests of bacterial OTU richness (*S*), diversity Exp(*H*), and community composition (redundancy analysis (RDA)) of floral nectar from both apple and pear, and sampling time including start of the flowering period (day 1), peak blooming (day 4), and three days later (day 7)

Fruit species	Model	*df*	test	*t* value	*p*‐value
Bacterial OTU richness					
Apple & Pear	Sampling time x species	2	*χ* ^2^	265.3	<.0001
Apple	Sampling time	2	*χ* ^2^	282.52	<.0001
Apple	Day 1 vs. Day 4	1	*χ* ^2^	235.08	<.0001
Apple	Day 1 vs. Day 7	1	*χ* ^2^	68.13	<.0001
Apple	Day 4 vs. Day 7	1	*χ* ^2^	45.6	<.0001
Pear	Sampling time	2	*χ* ^2^	141.15	<.0001
Pear	Day 1 vs. Day 4	1	*χ* ^2^	5.42	.02
Pear	Day 1 vs. Day 7	1	*χ* ^2^	33.36	<.0001
Pear	Day 4 vs. Day 7	1	*χ* ^2^	141.14	<.0001
Exp(*H*)					
Apple & Pear	Sampling time x species	2	*χ* ^2^	190.06	<.0001
Apple	Sampling time	2	*χ* ^2^	121.69	<.0001
Apple	Day 1 vs. Day 4	1	*χ* ^2^	104.31	<.0001
Apple	Day 1 vs. Day 7	1	*χ* ^2^	13.74	.0002
Apple	Day 4 vs. Day 7	1	*χ* ^2^	38.37	<.0001
Pear	Sampling time	2	*χ* ^2^	151.34	<.0001
Pear	Day 1 vs. Day 4	1	*χ* ^2^	1.67	.2
Pear	Day 1 vs. Day 7	1	*χ* ^2^	50.78	<.0001
Pear	Day 4 vs. Day 7	1	*χ* ^2^	149.86	<.0001
Bacterial community composition					
Apple & Pear	Sampling time x species	2	*F*	3.81	.001
Apple	Sampling time	2	*F*	8.64	.001
Apple	Day 1 vs. Day 4	1	*F*	12.58	.003
Apple	Day 1 vs. Day 7	1	*F*	9.98	.003
Apple	Day 4 vs. Day 7	1	*F*	1.57	.096
Pear	Sampling time	2	*F*	2.12	.001
Pear	Day 1 vs. Day 4	1	*F*	1.34	.03
Pear	Day 1 vs. Day 7	1	*F*	1.97	.003
Pear	Day 4 vs. Day 7	1	*F*	2.72	.003
Mantel test*					
Apple & Pear			*R*	0.06	.069
Apple			*R*	0.17	.014
Pear			*R*	−0.04	.776

* For the Mantel test, the correlation coefficient was given and not the *t* value.

**Figure 2:**
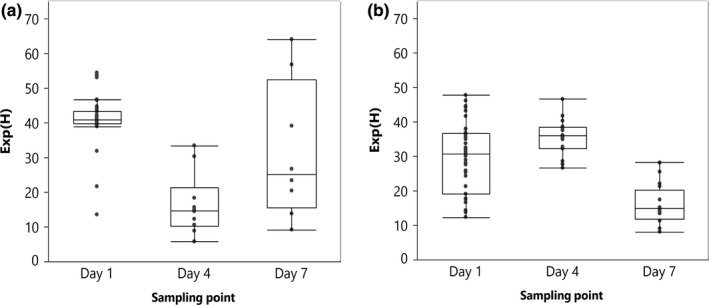
Exponentially transformed Shannon index [Exp(*H*)] of the bacterial communities inhabiting floral nectar of apple (a) (59 samples) and pear (b) (71 samples) over three time points, including start of the flowering period (day 1), peak blooming (day 4), and three days later (day 7). Outlier box plots represent the distribution of the samples around the median (horizontal line within the box). The first and third quartiles are the ends of the box

Exp(*H*) per sample varied between 5.8 and 64.1 (average: 35.8 ± 13.5) and between 8.1 and 47.8 (average: 28.2 ± 10.8) for apple and pear, respectively. The sampling time x species interaction for Exp(*H*) was also significant (*χ* ^2^ = 190.1, *df*: 2, *p* < .0001; Table 1, Figure 2). After splitting the dataset for both fruit species, sampling time was significant for both apple and pear (*χ* ^2^ = 121.7, *df*: 2, *p* < .0001; *χ* ^2^ = 151.3, *df*: 2, *p* < .0001, respectively). Within apple nectar, Exp(*H*) at day 1 (41.3 ± 7.1) was significantly higher compared to both day 4 (16.5 ± 8.9) and day 7 (31.7 ± 20.0) (*p* < .0001, *p* = .0002, respectively), while Exp(*H*) at day 4 was lower in comparison with day 7 (*p* < .0001). For the nectar of pear, the highest Exp(*H*) values were found for day 4 (35.6 ± 5.2) which were significantly higher compared to day 7 (16.1 ± 5.7) (*p* < .0001), but not compared to day 1 (29.5 ± 10.3) (*p* = .2). Exp(*H*) at day 1 was significantly higher compared to day 7 (*p* < .0001).

### 3.2 Bacterial community composition

**Figure 3:**
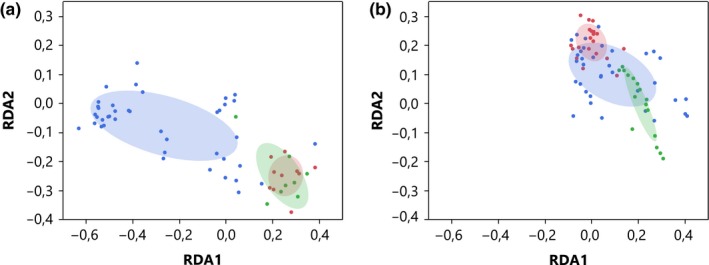
Redundancy analysis (RDA) plot (based on Hellinger distances) of the bacterial community composition found in floral nectar of apple (a) (59 samples) and pear (b) (71 samples) over three time points, including start of the flowering period (day 1, blue dots), peak blooming (day 4, red dots), and three days later (day 7, green dots). Confidence ellipses represent a bivariate normal density ellipse with coverage percentage of 50%

**Figure A2:**
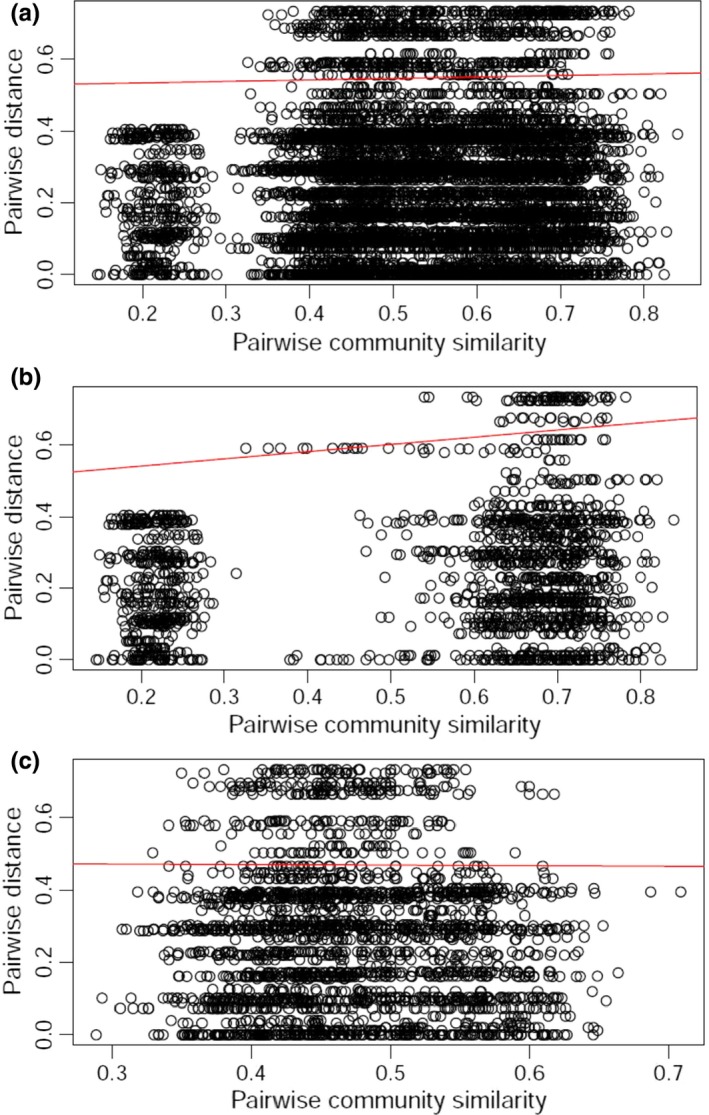
Relationship between bacterial community composition (pairwise community similarity) and orchard distance (pairwise distance) for both apple and pear together (A) and separately for apple (B) and pear (C)

Redundancy analysis showed a significant sampling time x species interaction (*F* = 3.8, *df*: 2, *p* = .001; Table 1, Figure 3). Splitting the dataset for pear and apple showed a significant sampling time effect on the bacterial community composition (*F* = 8.6, *df*: 2, *p* = .001; *F* = 2.1, *df* 2, *p* = .001, respectively). The bacterial community composition of apple nectar on day 1 differed significantly from the ones at day 4 and day 7 (*F* = 12.6, *df*: 1, *p* = .003; *F* = 10.0, *df*: 1, *p* = .003, respectively), but composition of the bacterial community at days 4 and 7 was similar (*F* = 1.6, *df*: 1, *p* = .1). For the floral nectar of pear, the bacterial community composition differed significantly between all three sampling points (Table 1). The Mantel test yielded no significant relation between nectar bacterial community composition similarity and orchard distance when data for the apple and pear orchards were lumped together (*R* = .1, *p* = .1; Table 1; Figure A2). This was not unexpected, because orchards controlled by the same grower were approximately less than 1 km apart. When both fruit species were tested separately, there was a significant relation between nectar bacterial community similarity and distance for apple orchards (*R* = .2, *p* = .01), but not for the pear orchards (*R *= −.04, *p* = .8; Figure A2).

### 3.3 Taxonomic classification

**Figure 4:**
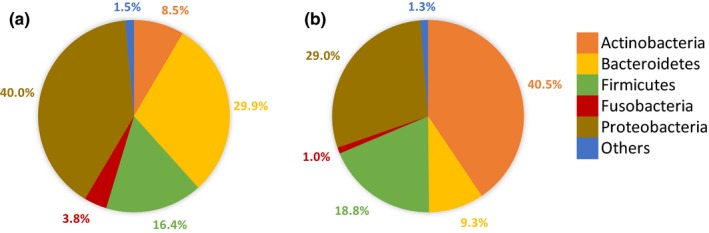
Distribution of bacterial phyla in floral nectar from apple (a) (59 samples) and pear (b) (71 samples), irrespective of sampling point. In total, 313 OTUs were found in apple nectar, 283 OTUs in pear nectar

The majority of the OTUs found belonged to the phylum Proteobacteria (34.0% of all sequences; 122 OTUs), followed by Actinobacteria (26.0%; 37 OTUs), Bacteroidetes (18.6%; 77 OTUs), Firmicutes (17.7%; 67 OTUs), and Fusobacteria (2.3%; 6 OTUs) (Table S3). The remaining fraction of sequences (*c.* 1.4%) could be attributed to Acidobacteria (2 OTUs), Armatimonadetes (1 OTU), Chlamydiae (2 OTUs), Chloroflexi (2 OTUs), Cyanobacteria (6 OTUs), Planctomycetes (2 OTUs), Tenericutes (2 OTUs), and Verrucomicrobia (1 OTU). Actinobacteria were considerably more abundant in the nectar of pear (40.5% of sequences) compared to the nectar of apple (8.5%), whereas Bacteroidetes, Proteobacteria, and Fusobacteria were more abundant in apple nectar (29.9%, 40.0%, and 3.8%, respectively) compared to pear nectar (9.3%, 29.0% and 1.0%, respectively; Figure 4). Firmicutes were more or less equally abundant in both fruit species (Apple: 16.4%; Pear 18.8%; Figure 4).

**Figure 5:**
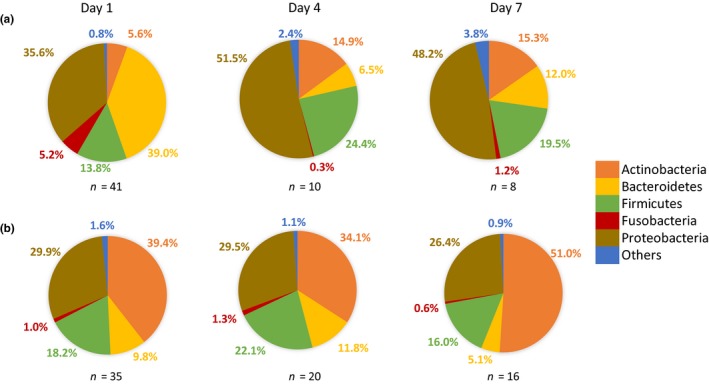
Distribution of phyla in floral nectar from apple (a) and pear (b) over three sampling points, including start of the flowering period (day 1), peak blooming (day 4), and three days later (day 7). Sequences were attributed to 270, 158, and 210 OTUs in apple nectar and 263, 244, and 205 OTUs in nectar from pear at day 1, day 4, and day 7, respectively. *n,* number of samples investigated

In apple nectar, the distribution of bacterial phyla in floral nectar clearly changed over time (Figure 5). At day 1, 39.0% and 5.2% of the sequences could be attributed to Bacteroidetes and Fusobacteria, respectively. At day 4 and day 7, this proportion decreased to 6.5% and 12% for Bacteroidetes and to 0.3% and 1.2% for Fusobacteria, respectively. The relative abundance of Actinobacteria increased during flowering from 5.6% at day 1 to 14.9% at day 4 and 15.3% at day 7. Furthermore, relative abundance of Firmicutes and Proteobacteria in apple nectar was 13.8% and 35.6% at day 1, 24.4% and 51.5% at day 4, and 19.5% and 48.2% at day 7. Such changes in relative abundance of phyla over time were less pronounced in pear nectar (Figure 5). Accordingly, the relative abundance of Actinobacteria decreased from 39.4% at day 1 to 34.1% at day 4, and increased again to 51.0% at day 7. Relative abundance of Bacteroidetes and Firmicutes changed from 9.8% and 18.2% at day 1 to 11.8% and 22.1% at day 4 and 5.1% and 16.0% at day 7. The relative abundance of the other phyla stayed relatively the same during the sampling period.

**Figure 6:**
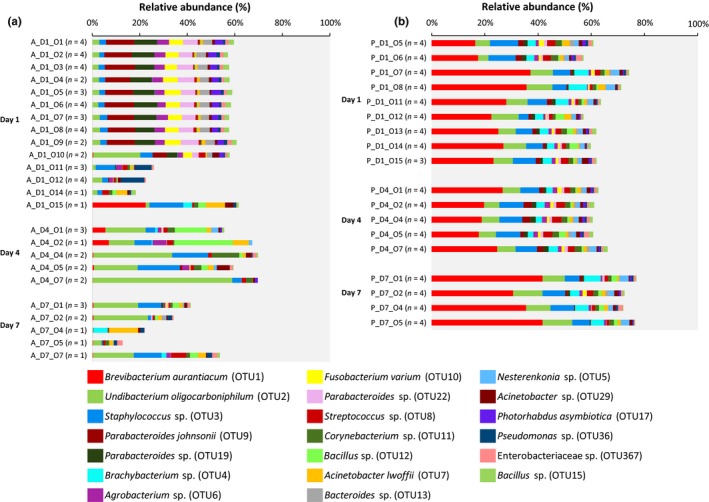
Relative abundance (%) of the 20 most abundant OTUs (both fruit species considered together) in floral nectar from apple (a) and pear (b) over three sampling points, including start of the flowering period (day 1), peak blooming (day 4), and three days later (day 7). Data are grouped per orchard; the number of trees sampled is given between brackets. OTUs were identified by a BLAST search against type materials in GenBank. Identification up to the species level is given when only 1 top hit was obtained. For identity percentages with GenBank entries, see Table 2. Sample ID: A: apple, P: pear, D: day, O: orchard

**Figure A3:**
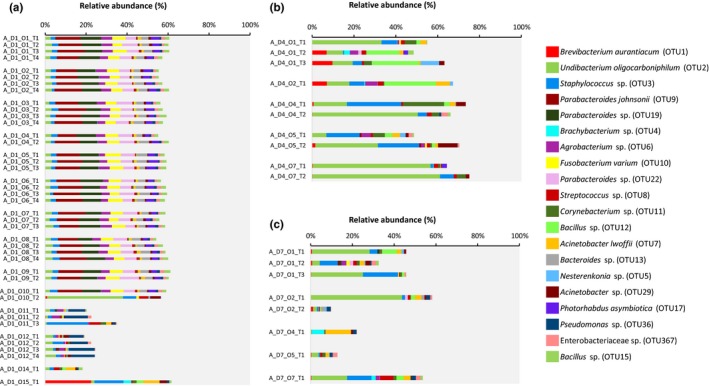
Relative abundance (%) of the 20 most abundant OTUs (both fruit species considered together) in floral nectar from apple over three sampling days, including start of the flowering period (day 1, A), peak blooming (day 4, B), and three days later (day 7, C). Data are shown for every sample investigated in this study. OTUs were identified by a BLAST search against type materials in GenBank. Identification up to the species level is given when only 1 top hit was obtained. For identity percentages with GenBank entries, see Table 2. Sample ID: A: apple, D: day, O: orchard

**Figure A4:**
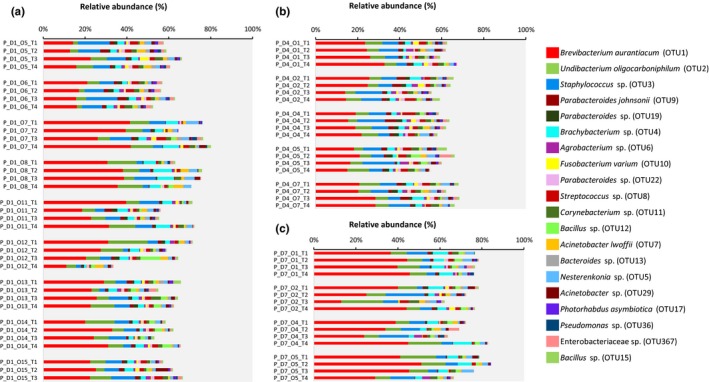
Relative abundance (%) of the 20 most abundant OTUs (both fruit species considered together) in floral nectar from pear over three sampling days, including start of the flowering period (day 1, A), peak blooming (day 4, B), and three days later (day 7, C). Data are shown for every sample investigated in this study. OTUs were identified by a BLAST search against type materials in GenBank. Identification up to the species level is given when only 1 top hit was obtained. For identity percentages with GenBank entries, see Table 2. Sample ID: P: pear, D: day, O: orchard

At lower taxonomic level, the bacterial communities of both apple and pear nectar were differently structured and changed differently over time (Figure 6; Table 2). Nectar samples from apple were commonly inhabited by *Undibacterium* (OTU2; Proteobacteria) (found in each investigated nectar sample of apple) and *Staphylococcus* (OTU3; Firmicutes) (Figure A3), reaching high relative abundances, especially at day 4 (28.2% and 9.3% for *Undibacterium* and *Staphylococcus*, respectively) and day 7 (15.4% and 5.5% for *Undibacterium* and *Staphylococcus*, respectively). At day 1, the nectar bacterial community was mainly dominated by OTUs corresponding to *Parabacteroides* (OTU9, OTU19, and OTU22; Bacteroidetes), *Agrobacterium/Rhizobium* (OTU6; Proteobacteria), and *Fusobacterium* (OTU10; Fusobacteria) reaching a total relative abundance of 9.0% for OTU9, 6.9% for OTU19, 4.7% OTU22, 3.6% for OTU6, and 4.6% for OTU10 (Figure 6; Table 2). Whereas at day 1 the bacterial community composition was highly similar for most of the samples (i.e., from apple orchards O1 until O9), a different community structure was observed at days 4 and 7 (Figure 6; Figure A3). Nectar of pear was strongly dominated by *Brevibacterium* (OTU1; *Actinobacteria*), *Undibacterium* (OTU2; Proteobacteria), and *Staphylococcus* (OTU3; Firmicutes), which were found in each investigated nectar sample of pear (Figure A4). These OTUs were found at a mean relative abundance of 28.3 ± 6.7% for OTU1, 8.0 ± 1.5% for OTU2, and 7.7 ± 0.5% for OTU3 over the three sampling points. In contrast to apple, the bacterial community composition was highly similar for all pear orchards over the three sampling points (Figure 6; Figure A4).

**Table 2:** Total relative abundance and prevalence of the 20 most abundant OTUs (both fruit species considered together) in floral nectar from apple (A) and pear (P) over three sampling days, including start of the flowering period (day 1), peak blooming (day 4), and three days later (day 7)

OTU	Taxonomic affiliation^a^			Taxonomic affiliation^b^	A						B					
					Day 1		Day 4		Day 7		Day 1		Day 4		Day 7	
	Phylum	Family	Species		Rel. ab. (%)^c^	Present in orchard (*n* = 14)	Rel. ab. (%)	Present in orchard (*n* = 5)	Rel. ab. (%)	Present in orchard (*n* = 5)	Rel. ab. (%)	Present in orchard (*n* = 9)	Rel. ab. (%)	Present in orchard (*n* = 5)	Rel. ab. (%)	Present in orchard (*n* = 4)
OTU1	Actinobacteria	Brevibacteriaceae	*Brevibacterium aurantiacum* (100%)	*Brevibacterium* sp. (100%) (nectar)	0.6%	9	2.7%	5	0.5%	5	25.9%	9	21.5%	5	37.4%	4
OTU2	Proteobacteria	Oxalobacteraceae	*Undibacterium oligocarboniphilum* (100%)	*Burkholderia* sp. (95%) (nectar)	3.7%	14	28.2%	5	15.4%	5	7.6%	9	6.5%	5	10.0%	4
OTU3	Firmicutes	Staphylococcaceae	Several *Staphylococcus* spp. (100%)	Unidentified bacterium (100%) (nectar) and several *Staphylococcus* spp*.,* including *Staphylococcus epidermidis* (100%)​ (honeybee stomach)	3.5%	14	9.3%	5	5.5%	5	7.2%	9	8.4%	5	7.4%	4
OTU9	Bacteroidetes	Tannerellaceae	*Parabacteroides johnsonii* (95%)	Uncultured bacterium (87%) (bee bread food stores)	9.0%	12	0.2%	1	0.1%	1	1.4%	9	2.3%	5	0.6%	4
OTU19	Bacteroidetes	Tannerellaceae	*Parabacteroides* sp. (96%) and *Parabacteroides chinchillae* (96%)	Uncultured bacterium (89%) (bee bread food stores)	6.9%	12	0.0%	0	0.3%	2	1.3%	9	1.6%	5	0.5%	4
OTU4	Actinobacteria	Dermabacteraceae	Several *Brachybacterium* spp*.* (100%)​	Unidentified bacterium (93%) (nectar) and *Janibacter sanguinis* (93%) (bee bread food stores)	0.1%	12	0.4%	3	1.0%	4	4.0%	9	2.7%	5	5.0%	4
OTU6	Proteobacteria	Rhizobiaceae	*Agrobacterium fabrum* (100%) and *Agrobacterium tumefaciens* (100%)	*Rhizobium pusense* (100%) (honeybee stomach) and *Agrobacterium tumefaciens* (100%) (honeybee stomach); Rhizobiaceae sp. (98%) (nectar)	3.6%	12	1.7%	4	0.5%	2	1.1%	9	1.7%	5	0.9%	4
OTU10	Fusobacteria	Fusobacteriaceae	*Fusobacterium varium* (100%)	Uncultured bacterium (95%) (bee bread food stores)	4.6%	12	0.2%	2	0.3%	1	0.9%	9	1.1%	5	0.5%	4
OTU22	Bacteroidetes	Tannerellaceae	Several *Parabacteroides* spp. (94%)​	Uncultured bacterium (88%) (bee bread food stores)	4.7%	11	0.2%	2	0.4%	3	0.7%	9	1.1%	5	0.2%	3
OTU8	Firmicutes	Streptococcaceae	Several *Streptococcus* spp. (100%)​	Uncultured bacterium (100%) (bee bread food stores)	1.0%	14	1.7%	5	1.5%	5	2.0%	9	3.0%	5	1.5%	4
OTU11	Actinobacteria	Corynebacteriaceae	*Corynebacterium accolens* (100%) and *Corynebacterium macginleyi* (100%)	*Corynebacterium* sp. (99%) (bee bread food stores)	0.4%	14	4.4%	5	0.8%	5	1.7%	9	2.2%	5	1.6%	4
OTU12	Firmicutes	Bacillaceae	Several *Bacillus* spp. (100%)	Several *Bacillus* spp*.,* including *Bacillus subtilis* (100%)​ (nectar)	0.3%	14	7.2%	4	1.8%	3	1.4%	9	1.1%	5	1.7%	4
OTU7	Proteobacteria	Moraxellaceae	*Acinetobacter lwoffii* (100%)	*Acinetobacter* sp. (99%) (nectar)	1.2%	14	2.1%	5	3.1%	5	1.6%	9	1.1%	5	1.2%	4
OTU13	Bacteroidetes	Bacteroidaceae	Several *Bacteroides* spp. (94%)​	Uncultured bacterium (86%) (bee bread food stores)	3.0%	11	0.0%	1	0.5%	2	0.7%	9	0.9%	5	0.4%	4
OTU5	Actinobacteria	Micrococcaceae	Several *Nesterenkonia* spp. (100%)​.	Unidentified bacterium (98%) (nectar) and *Nesterenkonia aethiopica* (98%) (honeybee stomach)	0.1%	3	1.3%	3	0.3%	3	2.0%	9	1.7%	5	2.6%	4
OTU29	Proteobacteria	Moraxellaceae	Several *Acinetobacter* spp. (99%)​	*Acinetobacter schindleri* (99%) (insect)	1.1%	14	2.0%	4	0.7%	4	1.1%	9	1.2%	5	1.1%	4
OTU17	Proteobacteria	Morganellaceae	*Photorhabdus asymbiotica* (94%)	*Aeromonas veronii* (91%) (alimentary tract midgut honeybee)	2.4%	10	0.5%	2	0.0%	0	0.5%	8	0.7%	5	0.4%	4
OTU36	Proteobacteria	Pseudomonadaceae	Several *Pseudomonas* spp. (100%)​	Unidentified bacterium (100%) (nectar)	2.4%	14	0.1%	2	1.3%	5	0.5%	9	0.6%	5	0.5%	4
OTU367	Proteobacteria	Enterobacteriaceae	Several Enterobacteriaceae spp. (99%)​	Several Enterobacteriaceae, including *Erwinia* sp. (99%)​ (nectar)	1.3%	13	0.6%	2	1.1%	5	1.0%	9	1.2%	5	0.9%	4
OTU15	Firmicutes	Bacillaceae	Several *Bacillus* spp. (100%)​.	Several *Bacillus* spp. (100%) (nectar)	1.7%	13	0.5%	3	0.3%	2	0.8%	9	1.4%	5	0.3%	4

a Nearest neighbor based on a BLAST search in GenBank against type strains. Percentage of sequence identity (on a total of 250 bp) is reported between brackets. Taxonomic assignment at phylum and family level was performed manually based on assigned species.

b Nearest neighbor based on a BLAST search in GenBank against sequence entries related to the nectar environment (search was limited to sequences associated with the keyword "nectar"). Percentage of sequence identity (on a total of 250 bp) is reported between brackets.

c Total relative abundance, calculated based on the total sum of sequences obtained for the OTU divided by the total number of sequences obtained at each sampling point for the apple or pear dataset x 100 (%).

## 4 DISCUSSION

### 4.1 Floral nectar of apple and pear differs in bacterial community composition

Our results clearly show that floral nectar of apple and pear harbors different microbial communities. Microbial communities were characterized using standard OTUs based on a threshold of 97% sequence similarity. This cutoff balances previous standards for defining bacterial species (Stackebrandt & Goebel, 1994) and a recognition of spurious diversity accumulated through PCR and sequencing errors (Acinas, Sarma‐Rupavtarm, Klepac‐Ceraj, & Polz, 2005; Kunin, Engelbrektson, Ochman, & Hugenholtz, 2010). However, there is a growing tendency to move toward analysis of exact sequence variants, also termed amplicon sequence variants (ASVs) (Callahan, McMurdie, & Holmes, 2017) or zero‐radius OTUs (zOTUs) (Edgar, 2016), increasing taxonomic resolution. Recent research has shown that both methods yield similar ecological conclusions for broad scale alpha‐ and beta‐diversity analyses (Glassman & Martiny, 2018), thereby reinforcing the use of any of these methods. In total, 327 bacterial OTUs were observed, and more than 80% of these OTUs were found in both apple and pear nectar. The bacterial OTU richness per sample was similar for apple and pear nectar, whereas the bacterial OTU diversity per sample was higher for apple nectar compared to pear nectar. In line with previous studies on nectar bacteria (Aizenberg‐Gershtein, Izhaki, & Halpern, 2013; Álvarez‐Pérez et al., 2012; Jacquemyn, Lenaerts, Tyteca, et al., 2013), Proteobacteria, Actinobacteria, and Firmicutes were commonly detected in nectar from both fruit species. Specifically, the nectar of pear was dominated by Actinobacteria, followed by Proteobacteria and Firmicutes. In comparison with pear nectar, apple nectar was strongly enriched in Bacteroidetes, especially at the cost of Actinobacteria. As far as we know, bacteria from the phylum Bacteroidetes have only been rarely found in nectar, more particularly in almond nectar only (Fridman et al., 2012; Schaeffer et al., 2017), but they have also been associated with the apple flower microbiome previously (Shade et al., 2013). Members of Bacteroidetes are strongly associated with animals and occur in the guts of, for example, honeybees and bumblebees (Engel & Moran, 2013; Koch & Schmid‐Hempel, 2011), which may transport and inoculate the bacteria into flower nectar. For both fruit species, floral nectar was dominated by only a few bacterial species. Nectar of pear was strongly dominated by a *Brevibacterium* OTU (OTU1; Actinobacteria), reaching a relative abundance of 37.4%, while apple nectar was dominated by a *Undibacterium* OTU (OTU2; Proteobacteria), especially as bloom progressed (reaching a relative abundance of 28.2%). Furthermore, the *Brevibacterium* and *Undibacterium* OTUs were found in every investigated nectar sample of pear and apple, respectively, suggesting a strong association between these OTUs and the nectar of pear or apple. *Brevibacterium* species were already found in nectar samples of the forest herb *Pulmonaria officinalis* L. (Jacquemyn, Lenaerts, Brys, et al., 2013), while *Undibacterium* species have not yet been found in nectar, unlike closely related *Burkholderia* species did (Álvarez‐Pérez et al., 2012). The exact mechanisms explaining the differences in microbial community composition between both fruit species, however, still remain to be unraveled. Nevertheless, it is likely to assume that the observed differences are caused by differences in nectar chemistry, which is known to play a key role in the assembly of microbial communities in nectar (Lievens et al., 2015). Sugar concentration is lower in pear nectar (*c.* 10%) than in apple nectar (*c.* 40%), and apple nectar contains more disaccharides, while pear nectar mainly consists of monosaccharides (Quinet et al., 2016). Additionally, large differences can be observed in pH between nectar from apple (*c*. pH 4) and from pear (*c*. pH 8) (Smessaert J., Honnay O. & Keulemans W., unpublished results). Further research is needed to truly explain the observed differences between both fruit species. This said, it should also be noted that when using an amplicon sequencing approach to characterize microbial diversity, one cannot be certain that all bacterial sequences detected represent bacteria that can actually grow and thrive in the harsh nectar environment, as they may also represent dead or inactive propagules (Wuyts et al., 2018).

### 4.2 Nectar bacterial community composition changes during flowering

Bacterial richness and diversity of both apple and pear nectar fluctuated during flowering. Whereas OTU richness and diversity decreased at day 4 and increased again at day 7 in apple nectar, the opposite occurred in pear nectar for both bacterial richness and diversity. By contrast, when analyzing the whole apple microbiome at different times throughout the flowering season, a clear successional pattern of microbial groups was observed whose abundances peaked at different times during bloom, with a clear increase in bacterial diversity after bud opening (Shade et al., 2013). We hypothesize that multiple mechanisms could be at play in driving temporal changes in nectar bacterial community composition, including environmental factors pollinator activity, and nectar chemical traits. Environmental factors such as temperature, humidity, and elevation are not only known to affect microbial metabolism (Fuhrman, 2009), but they may also affect nectar features (Lievens et al., 2015), and therefore also microbial community composition (Samuni‐Blank et al., 2014). Pollinators are important dispersal agents of microorganisms that can shape the microbial community composition of nectar by introducing microorganisms into the nectar. At the same time, they can get contaminated by bacteria that were already introduced in the nectar previously, which in turn can be dispersed to other flowers (Aizenberg‐Gershtein et al., 2013). In pome fruits, several studies have investigated the dispersal of *E. amylovora*, the causal agent of fire blight, and found significant relations with pollinators (Nuclo et al., 1998; Pusey, 2002; Van Laere et al., 1980). Furthermore, nectar characteristics such as the composition and concentration of sugars, amino acids, proteins, and secondary metabolites have been found to change over time and during flowering (Aizenberg‐Gershtein et al., 2015; Roy, Schmitt, Thomas, & Carter, 2017), which in turn may affect microbial community composition over time. However, as precise data in this regard are lacking for our study, we cannot confirm any of these scenarios yet.

### 4.3 Apple nectar bacterial community composition was also found to vary spatially

Next to temporal variation, also spatial variation was found in the bacterial community composition. Apple orchards showed a pattern of isolation by distance, while pear orchards did not. This may possibly be explained by the nectar features of both fruit species. It is known that apple nectar is more attractive for pollinators than pear nectar, likely due to higher sugar concentrations (Quinet et al., 2016). Therefore, pollinators might stay more local in apple orchards as they are highly rewarded by their flowers. As a result, microbes will be particularly vectored within the same orchard or orchards nearby, causing the nectar bacterial community to be more similar at short distances, but different at longer distances. Furthermore, it may be possible that the use of pesticides has affected the microbial community composition, as has been observed previously (Schaeffer et al., 2017). Pear is often more susceptible to fire blight than apple (Bonn & van der Zwet, 2000) and thus requires more intense disease management. Although we have no information about potential pesticide use in the orchards studied, it may be a good reason why the bacterial community composition in pear nectar was more similar, irrespective of geographical location.

### 4.4 Implications for apple and pear production

It is clear from our study that both apple and pear nectar contain bacteria. The presence of these bacteria in the nectar may influence pollinator attraction and hence indirectly influence fruit set, yield, and also fruit quality as pollinators have a positive influence on the final fruit quality of apples and pears (Geslin et al., 2017; Quinet et al., 2016). However, little is known so far about how the presence of bacteria in nectar attract or repel pollinators, and only a few bacterial species have been tested so far (Good et al., 2014; Junker et al., 2014; Vannette et al., 2013). Likewise, the ecological role of the bacterial species found in this study and others remains to be investigated. Furthermore, very little is known about the richness, diversity, and community composition of microbiota in the floral nectar of perennial fruit crops, despite their importance to ecosystem processes such as pollination and additionally fruit production. Further research might focus on the effects of bacteria on nectar quality, quantity, and odor, as well as on pollinator behavior, pollination efficacy, and fruit production for pome fruits as done previously for wild plants (Pozo et al., 2015). If we succeed in identifying useful bacterial species (e.g., bacteria that improve attraction of pollinators, especially a problem in pear), we could then start testing targeted manipulations or inoculations with certain bacteria. For instance, when virgin nectar could be inoculated with a useful bacterium, chances are high that they manage to become dominant and give other, later‐arriving species no chance to settle (Álvarez‐Pérez, Lievens, & Fukami, 2019; Peay, Belisle, & Fukami, 2011).

## CONFLICT OF INTERESTS

None declared.

## AUTHOR CONTRIBUTIONS

Jolien Smessaert, Olivier Honnay, Wannes Keulemans, and Bart Lievens involved in conceptualization and data curation; Jolien Smessaert, Maarten Van Geel, Sam Crauwels, and Olivier Honnay performed formal analysis; Olivier Honnay and Wannes Keulemans involved in funding acquisition; Jolien Smessaert and Christel Verreth involved in the investigation process; Jolien Smessaert, Olivier Honnay, and Wannes Keulemans administered the project; Jolien Smessaert visualized and wrote the original manuscript; and Olivier Honnay, Wannes Keulemans, and Bart Lievens supervised the project and wrote, reviewed, and edited the manuscript.

## ETHICS STATEMENT

None required.

## Data Availability

All sequence files are available from the Sequence Read Archive (BioProject accession PRJNA488015). All other relevant data are within the manuscript and its Appendices.
